# Supplementary material for: Regulation of IGF1R by MicroRNA-15b Contributes to the Anticancer Effects of Calorie Restriction in a Murine C3-TAg Model of Triple-Negative Breast Cancer
Source: Cancers (Basel). 2023 Aug 29;15(17):4320. doi: 10.3390/cancers15174320 (PMC10486801; doi:10.3390/cancers15174320)

MBA-MD-231 cells

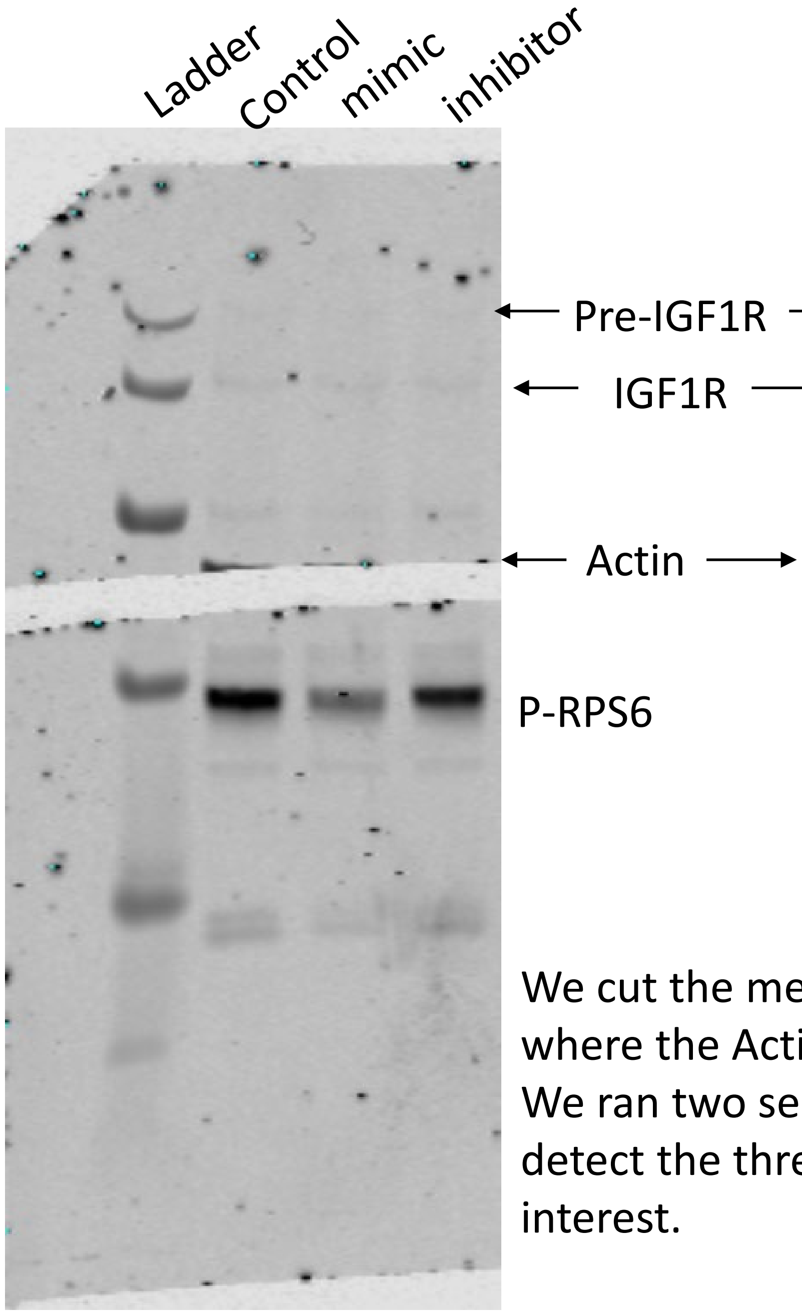

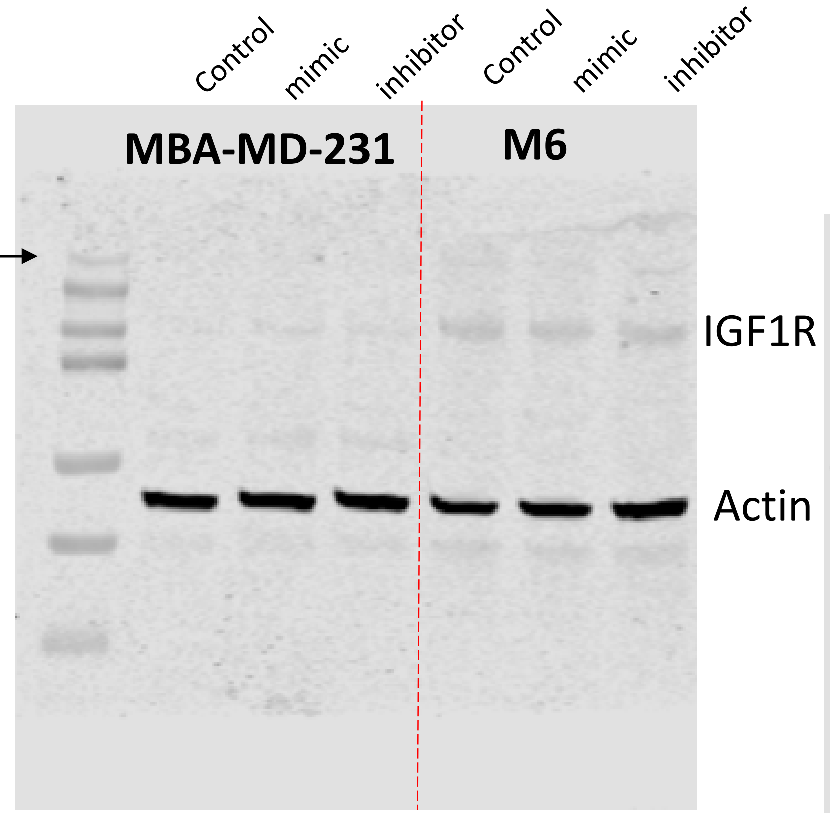

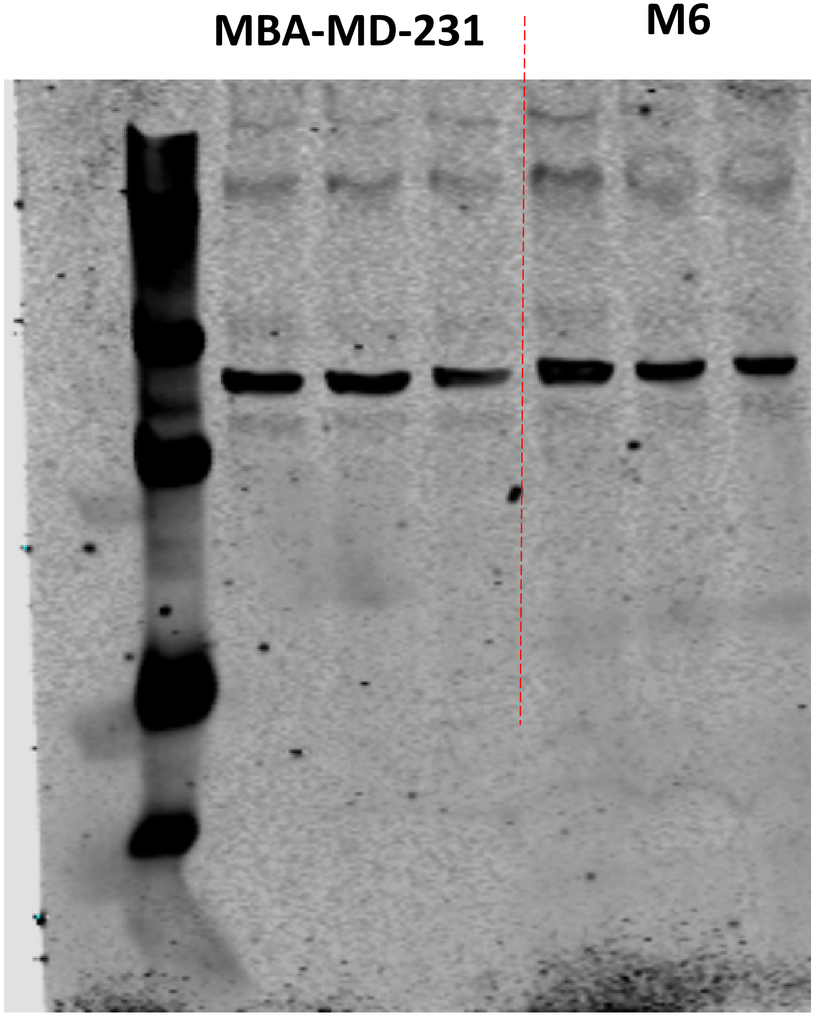

We cut the membrane right where the Actin bands were. We ran two separated gels detect the three proteins of interest.

M6 cells

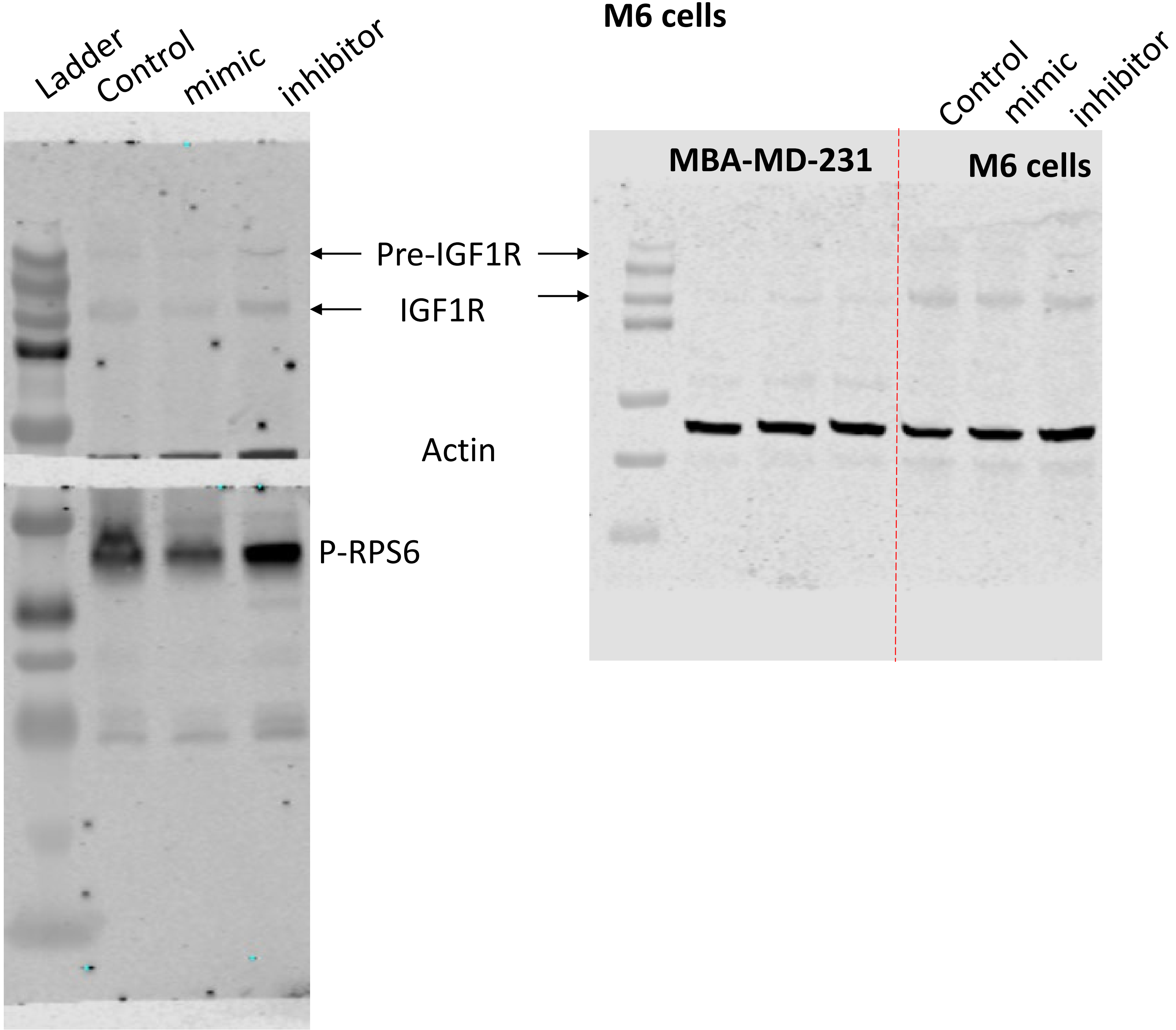

Ladder
Control
mimic
inhibitor
Pre-IGF1R
IGF1R
Actin
P-RPS6
Control
mimic
inhibitor
MBA-MD-231
M6 cells

M6C and M6C cells

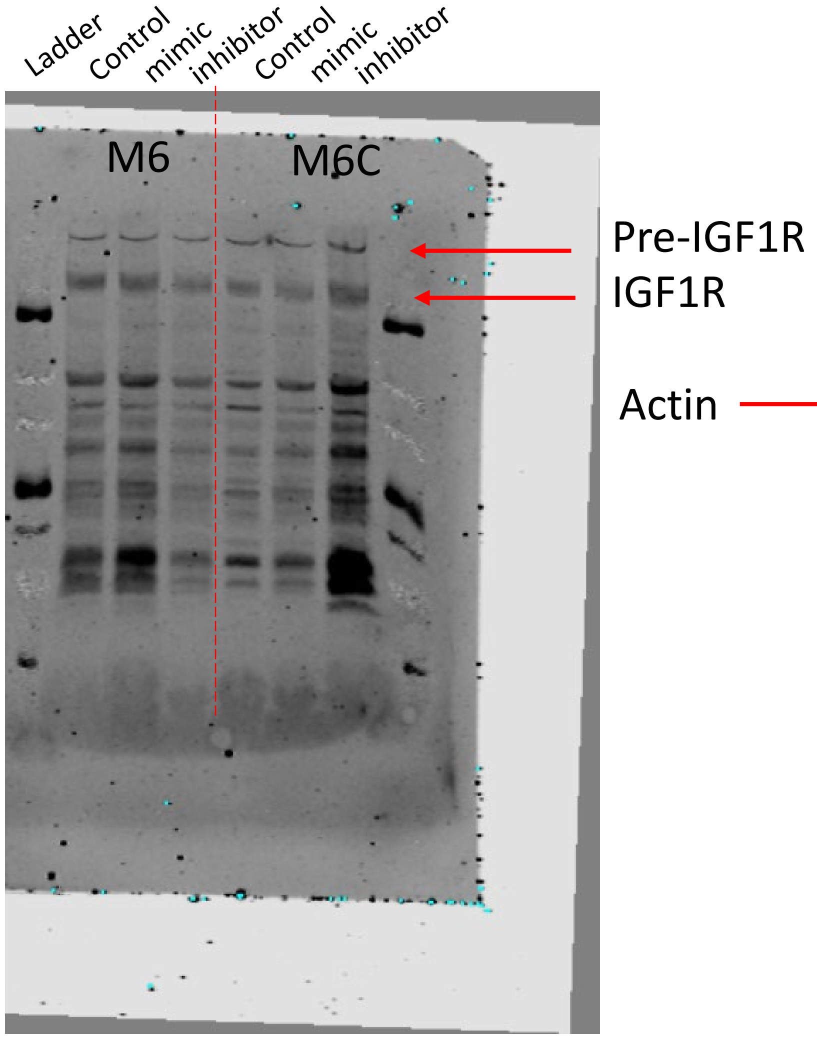

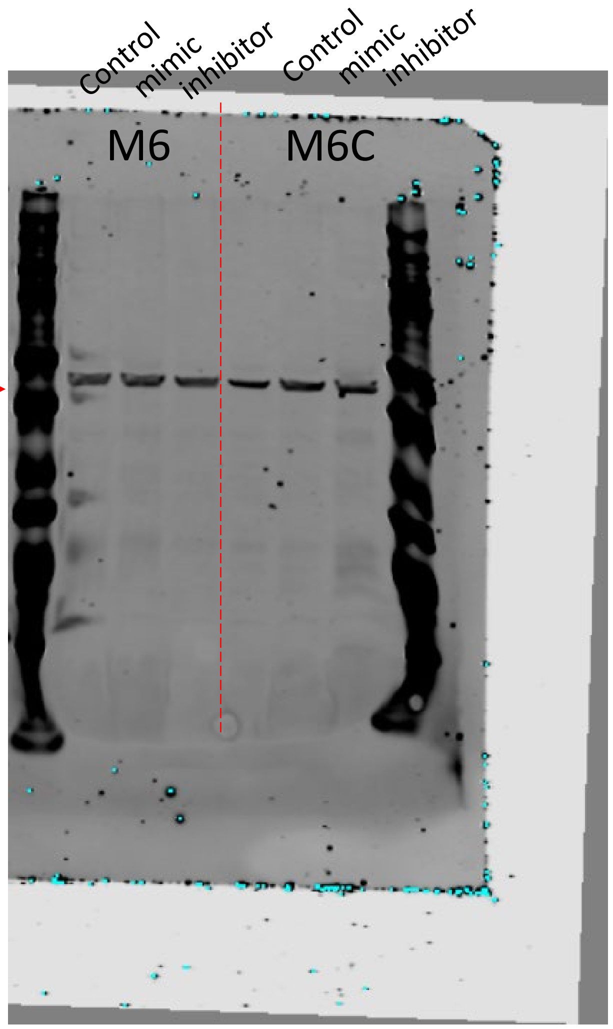

Repetition M6C cells cutting membrane to detect P-RPS6

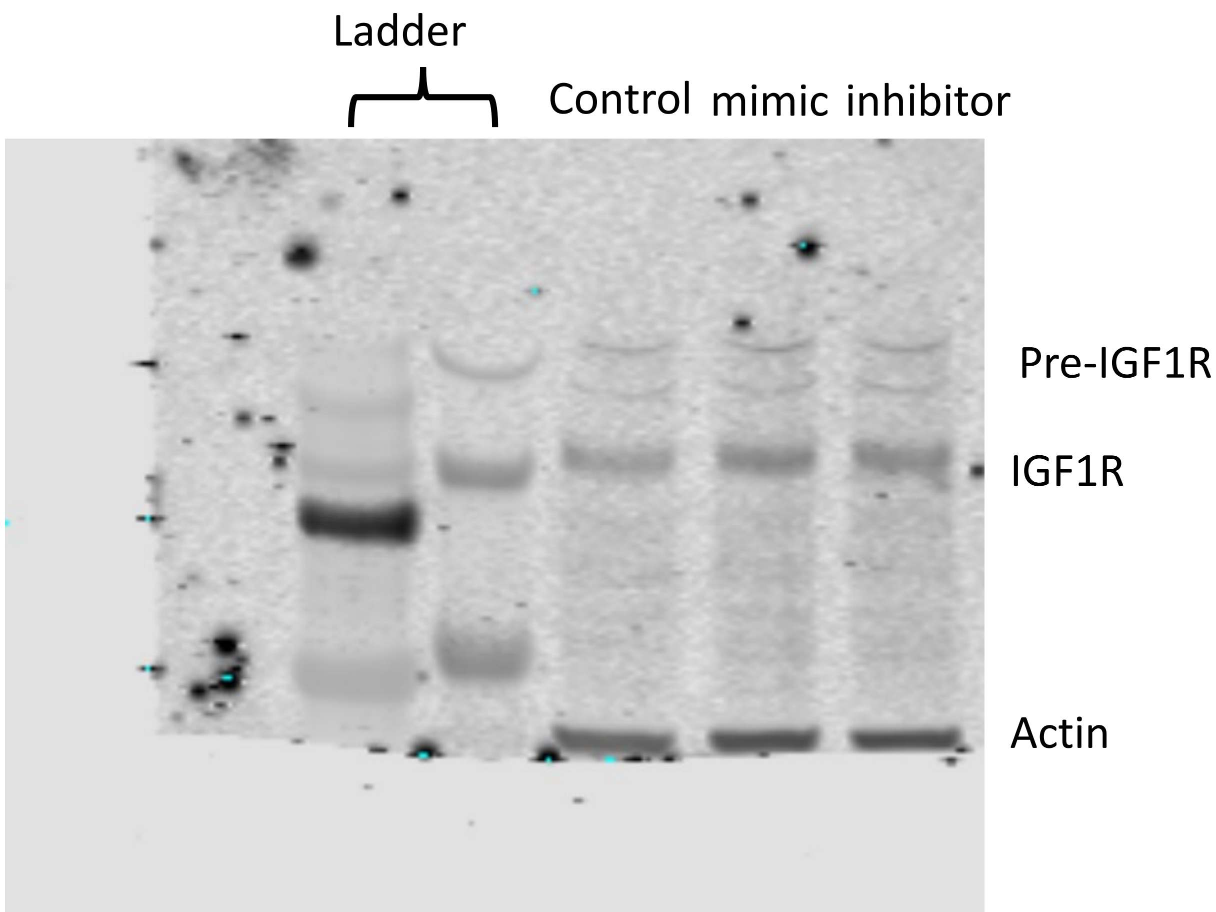

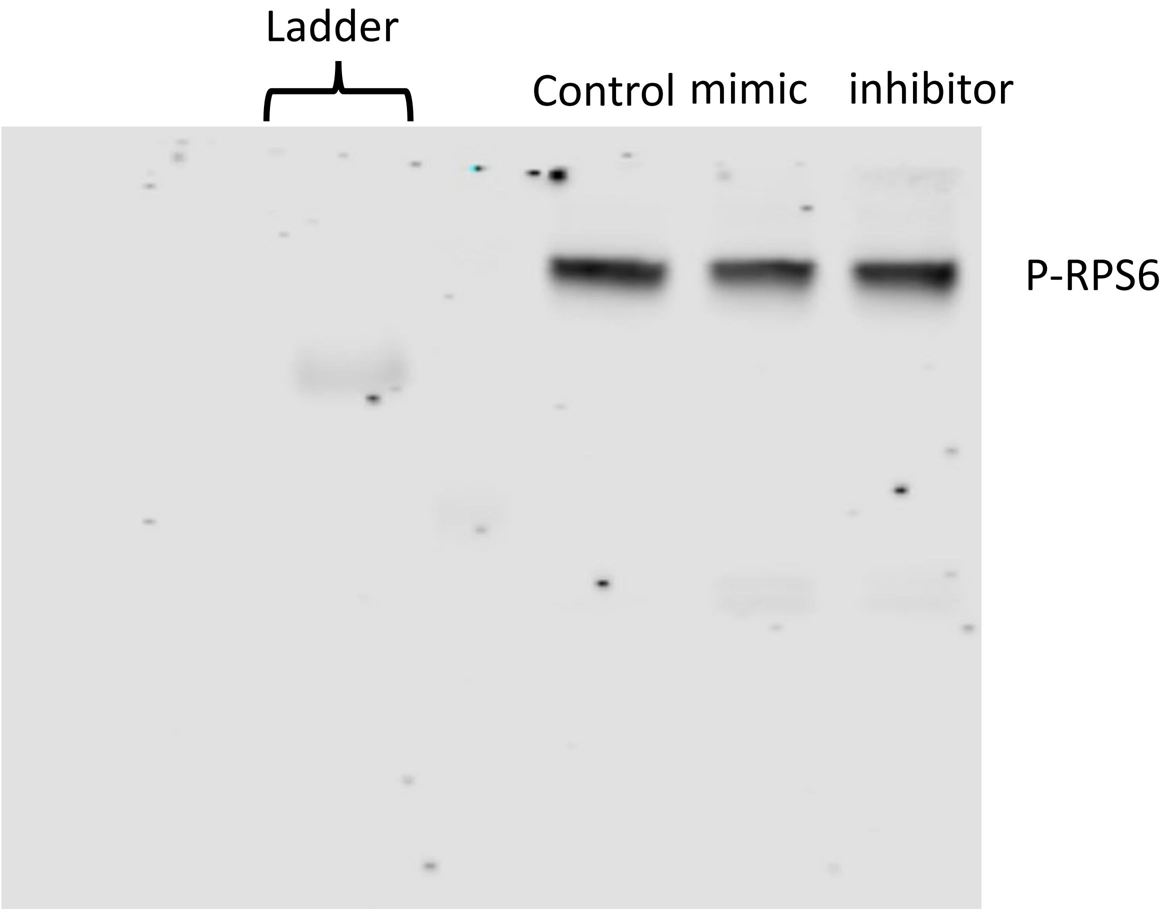

Supplement: Supplementary file 1 [file cancers-15-04320-s001.zip › cancers-2482927-origimages.pdf]
