# Supplementary material for: Regulation of IGF1R by MicroRNA-15b Contributes to the Anticancer Effects of Calorie Restriction in a Murine C3-TAg Model of Triple-Negative Breast Cancer
Source: Cancers (Basel). 2023 Aug 29;15(17):4320. doi: 10.3390/cancers15174320 (PMC10486801; doi:10.3390/cancers15174320)
Supplement: Supplementary file 1 [file cancers-15-04320-s001.zip › cancers-2482927-supplementary-english.pdf]

Supplementary Figures

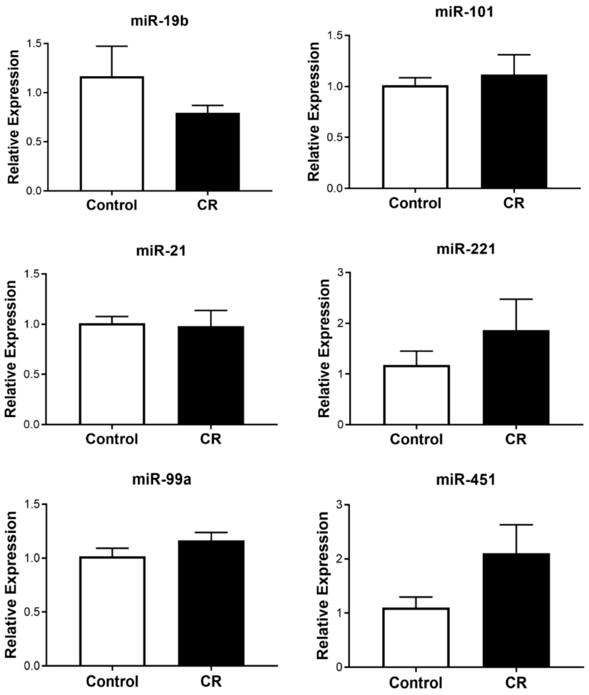

Figure S1: Expression of miRNAs associated with IGF1/mTOR signaling. Gene expression analysis by qRT-PCR of individual miRNAs in control diet (n =5) and CR secondary tumors (n =7). Values graphed are mean ± SEM; *, $p < 0.05$.

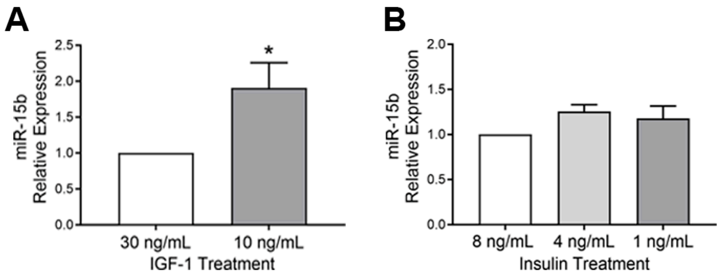

Figure S2: Differential effect of IGF1 and insulin on miR-15b expression in M6 cells. Gene expression analysis by qRT-PCR of miR-15b following (A) IGF1 treatment and (B) insulin treatment in M6 cells. Values graphed are mean ± SEM;*, $p < 0.05$.

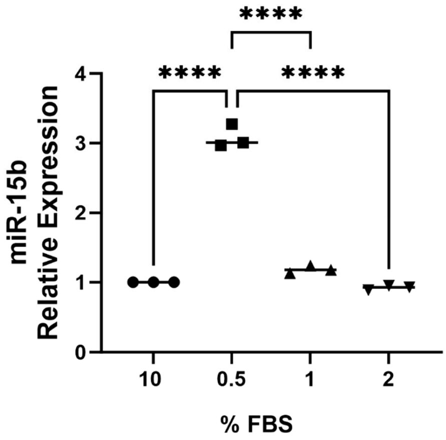

Figure S3: Effects of serum restriction on miR-15b expression. M6 cells were cultured in media supplemented with 10% FBS, followed by 4 hours in serum-free media and subsequently cultured for 18 hours in media supplemented with 0.5%, 1%, or 2% FBS. The expression of miR -15b was evaluated using qPCR. Differences were analyzed using one-way ANOVA, followed by Tukey's post hoc test. (*: $p < 0.05$, **: $p < 0.01$, ***: $p < 0.001$).

Commented [MEE1]: Please check if meaning is retained.

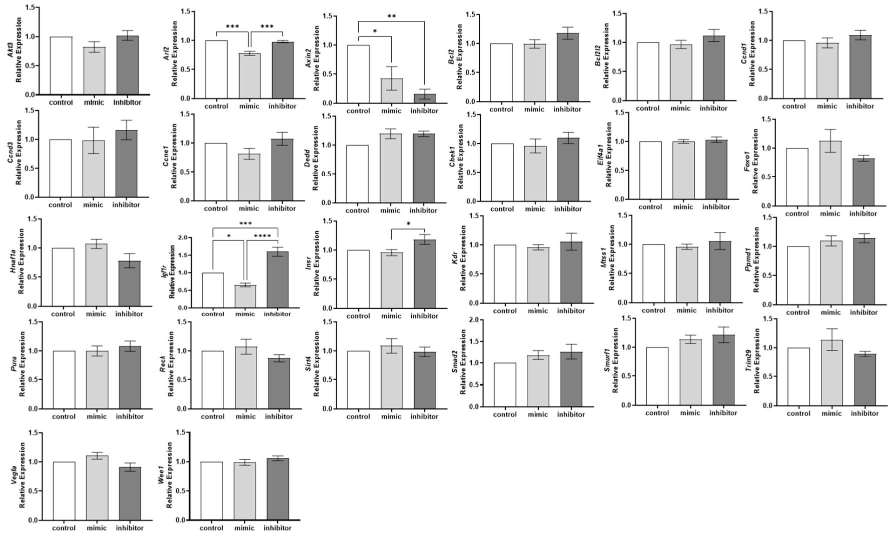

Figure S4: Expression analysis of miR-15b predicted target in M6 cell. qPCR analysis of the 26 predicted target genes in M6 cells expressing an miR-15b mimic (mimic) or a miR-15b inhibitor (inhibitor). Each bar represents the mean ± SEM. Differences were analyzed using one-way ANOVA, followed by Tukey's post hoc test. (*: P< 0.05, **: P < 0.01, ***: P < 0.001 relative to control cells).

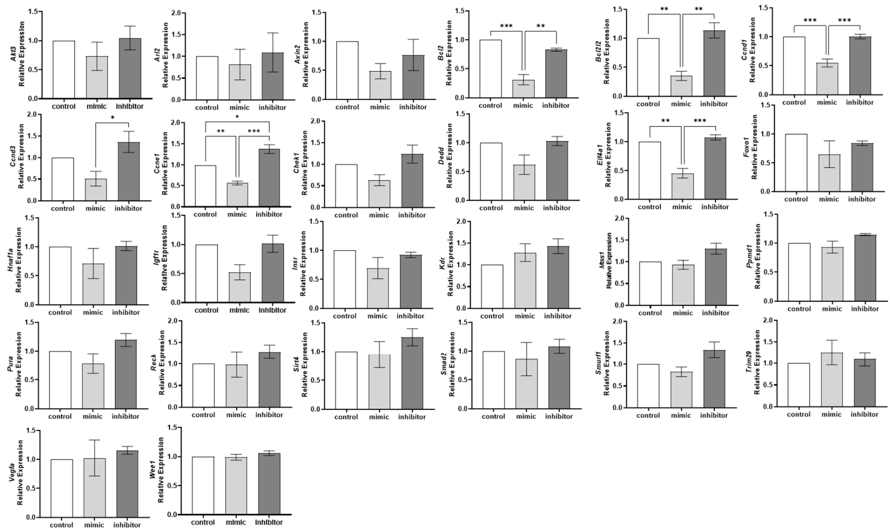

Figure S5: Expression analysis of miR-15b predicted target in MDA-MB-231 cells. qPCR analysis of the 26 predicted target genes in MDA-MB-231 cells expressing an miR-15b mimic (mimic) or an miR-15b inhibitor (inhibitor). Each bar represents the mean ± SEM. Differences were analyzed using one-way ANOVA, followed by Tukey's post hoc test. (*: $p < 0.05$, **: $p < 0.01$, ***: $p < 0.001$ relative to control cells).

Supplementary Tables

Table S1: Primers used for qPCR analysis using SYBR® Green

Target gene	Gene Name	Gene function	Primers	Product size (bp)
AKT3	serine/ threonine kinase 3	Regulator of PI3K/AKT/mTOR and CCR5 pathways.	F: TGGGTTCAGAAGAGGGGAGAA<br>R:AGGGGATAAGGTAAGTCCACATC	122
ARL2	ADP ribosylation factor like GTPase 2	Regulates microtubuless dynamics of and mitochondrial functions.	F: CTTGGACAACGCTGGCAAAAC<br>R: GCCATCCGTGCTCTCAAAGTA	190
AXIN2	Axin-related protein 2	Rregulates the stability of beta-catenin in the Wnt signaling pathway.	F: CGGCTGCGCTTTGATAAGG<br>R: ATGTGAGCCTCCTCTCTTTTACA	h: 148<br>m: 158
BCL2	BCL2 Apoptosis Regulator	Suppressor of apoptosis.	F: ATGCCTTTGTGGAACTATATGGC<br>R: GGTATGCACCCAGAGTGATGC	120
BCL2L2	BCL2 Like 2	Suppressor of apoptosis.	F: GCGGAGTTCACAGCTCTATAC<br>R: AAAAGGCCCCTACAGTTACCA	136
CCND1	Cyclin D1	Ccell cycle, G1/S transition.	h F: GAAGGAGACCATCCCCCTGA<br>h R: GAAATCGTGCGGGGTCATTG	142
			m F: CAGCCCCAACAACTTCCTCTC<br>m R: CTTGTTTAGCCAGAGGCCGGT	106
CCND3	Cyclin D3	Ccell cycle G1/S transition.	F: CTGGATGCTGGAGGTGTGTG<br>R: CTCAATAGTCAGGGGCGTGG	181
CCNE1	Cyclin E1	Ccell cycle G1/S transition.	h F: CCCATCATGCCGAGGGAG<br>h R: TATTGTCCCAAGGCTGGCTC	199
			m F: GTGGCTCCGACCTTTCAGTC<br>m R: CACAGTCTTGTCAATCTTGGCA	101
CHEK1	Checkpoint Kinase 1	Cell cycle arrest	h F: TGCGTTGTAAGATTTATTTTGGCTC<br>h R: ATGCTTTACTTCAGCCCGGT	122
			m F: CAAAGGACTGCTTGTCGCTG<br>m R: TCTATGGCCCGCTTCATGTC	171
DEDD	Death Effector Domain Containing	Ppro-apoptosis.	F: ACCGCATGTTCGACATCGT<br>R: CACGTCCATTTCGGATGAGTC	117
EIF4A1	Eukaryotic Translation Initiation Factor 4A1	Rregulates initiation of protein translation.	F: ATGTCTGCGAGTCAGGATTCT<br>R: AGCTATCCACAATCTCGTTCCA	100
FOXO1	Forkhead Box Protein O1	Regulates metabolic homeostasis in response to oxidative stress.	F: CCCAGGCCGGAGTTTAACC<br>R: GTTGCTCATAAAGTCGGTGCT	h:138<br>m :132

Continuation supplementary Table 1

HNF1A	Hepatocyte Nuclear Factor 1-Alpha	Transcription factor required for the expression of several liver-specific genes.	h F:CAGAGCCATGTGACCCAGAG	181
			h R: GCTTGGTGGGCGTGAGG	
			m F:AGTTGCCTAATGGCCTTGGA	165
			m R:GCCATGGGTCCTCCTGAAGAA	
IGF1R	Insulin-Like Growth Factor 1 Receptor	B~~b~~inds insulin-like growth factor with a high affinity.	F: CATGTGCTGGCAGTATAACCC	129
			R: TCGGGAGGCTTGTTCTCCT	
INSR	Insulin Receptor	A~~a~~ctivates the insulin signaling pathway.	h F: CGGCGATATGGTGATGAGGA	167
			h R: ACGTAGAAATAGGTGGGTTCCG	
			m F: ATGGGCTTCGGGAGAGGAT	121
			m R: GGATGTCCATACCAGGGCAC	
KDR	Kinase insert domain receptor	E~~e~~ncodes ~~a~~ VEGF receptors ~~of the~~	h F: GACTGAATGCGGGAGGTTCA	120
			h R: AATACTGATGGGTTGCGGGG	
			m F: TTTGGCAAATACAACCCTTCAGA	133
			m R: GCAGAAGATACTGTCACCACC	
MTSS1	MTSS I-BAR domain Containing 1	Metastasis s~~S~~uppressor.	F: ATGGAGGCTGTGATCGAGAAG	114
			R: TCCGGCTTTGTTTATGAAGTCTT	
PPM1D	protein phosphatase, Mg^{2+}/Mn^{2+} dependent 1D	S~~s~~uppresses p53-mediated transcription and apoptosis.	F: GACTCAGGGGTGGTTCTTGG	182
			R: GTGAGTCGAGGTCGTTTCCA	
			F: GATGTATGTAGCGCATGTAGGTG	110
			R: GTTCTGGCTTGTGATCTTGTGT	
PURA	Purine Rich Element Binding Protein A	C~~c~~ontrol of both DNA replication and transcription.	F: ATCCGCCAGACAGTCAACC	128
			R: TCCACTCCATAGTCGTCGATG	
RECK	reversion inducing cysteine rich protein with kazal motifs	S~~s~~uppressor of metastasis, negative regulator for matrix metalloproteinase-9.	h F: ACCGTCTCAGCTATTTGCAG	167
			h R: TAAGCCAACCCAGCCATCAG	
			m F: ATGTGGCACCTCTTAGCACC	153
			m R: ACTGAGCGAGTGTGAACTGG	
SIRT4	Sirtuin 4	R~~r~~egulates insulin secretion.	h F: TTCTCCTCCCACCAGCCTAA	174
			h R: CCCACAATCCAAGCACAGGA	
			m F: AATCTTGGCGTGGGAGACAC	147
			m R: CCGCTCATTCTTATTCTTCCACA	

Commented [MEE2]: Please check if there is missing information.

Continuation supplementary Table 1

SMAD2	SMAD Family Member 2	M~~m~~ediates transforming growth factor (TGF)-beta signaling.	F: ATGTCGTCCATCTTGCCATTC	173
			R: AACCGTCCTGTTTTCTTTAGCTT	
SMURF1	SMAD Specific E3 Ubiquitin Protein Ligase 1	~~Roll~~ Role in ~~the~~ regulating cell motility, cell signaling, and cell polarity.	h F: CCCGCTCCAAGGCTTCAA	109
			h R: AAAGCAGGTATGGGCCTTCG	
			m F: AGCATCAAGATCCGTCTGACA	104
			m R: CCAGAGCCGTCCACAACAAT	
TRIM29	Tripartite Motif Containing 29	T~~t~~ranscriptional regulatory factor involved in differentiation and~~&~~ carcinogenesis.	h F: GGGTGGAGGAGATAGGCAGA	159
			h R: TGCAATGACAGCTCCGTCTC	
			m F: AGAATGGCACTAAAGCAGACAG	122
			m R: AAATAGGCCACTCTTCCCCTC	
VEGFA	Vascular Endothelial Growth Factor A	P~~p~~romotes angiogenesis.	h F: TGCCCGCTGCTGTCTAATG	137
			h R: GCGAGTCTGTGTTTTTGCAG	
			m F: CCCGGGCCTCGGTTC	198
			m R: CCTGGGACCACTTGGCAT	
WEE1	WEE1 G2 Checkpoint Kinase	N~~n~~egative regulator of entry into mitosis (G2/ M transition).	h F: ATGGGCCTCGTCTGGAACTT	189
			h R: GCATCCTATGGCTCGGGAGT	
			m F: GTCGCCCGTCAAATCACCTT	249
			m R: GAGCCGGAATCAATAACTCGC	

h: human, m: mouse

Table S2: Calorie restriction slows lesion progression in mammary ductal epithelium in a model of basal-like breast cancer.

Ductal Density						
	Low		Medium		High	
**Week**	**Control**	**CR**	**Control**	**CR**	**Control**	**CR**
8	-	1 (14.3%)	5 (62.5%)	6 (85.7%)	3 (37.5%)	-
11	-	5 (62.5%)	5 (62.5%)	3 (37.5%)	3 (37.5%)	-
14	1 (12.5%)	2 (28.6%)	4 (50%)	5 (71.4%)	3 (37.5%)	-
17	1 (12.5%)	5 (62.5%)	2 (25%)	3 (37.5%)	5 (62.5%)	-
20	-	5 (62.5%)	1 (12.5%)	3 (37.5%)	7 (87.5%)	-

Major Lesion Grade								
	Normal		AH		MIN		IDC	
**Week**	**Control**	**CR**	**Control**	**CR**	**Control**	**CR**	**Control**	**CR**
8	5 (62.5%)	6 (85.7%)	3 (37.5%)	-	-	1 (14.3%)	-	-
11	1 (12.5%)	7 (87.5%)	4 (50%)	1 (12.5%)	3 (37.5%)	-	-	-
14	1 (12.5%)	3 (42.9%)	1 (12.5%)	3 (42.9%)	6 (75%)	1 (14.9%)	-	-
17	1 (14.9%)	7 (87.5%)	1 (14.9%)	1 (12.5%)	5 (71.4%)	-	-	-
20	-	5 (62.5%)	-	-	7 (87.5%)	3 (37.5%)	1 (12.5%)	-

Table S3: Insulin-like growth factor-1-signaling--pathway-targeting miRNAs.

Targeting miRNA	Species	Reference
miR-15b	human	(1), (2)
miR-19b	human	(3)
miR-21	human, pig, mouse	(4), (5), (6)
miR-99a	human	(7), (8), (9)
miR-101	rat	(10), (11)
miR-221	human, mouse	(12, 13)
miR-451	mouse	(14, 15)
miR-199a	human	(16)
miR-199a-5p	Human, rat, mouse	(17), (18)
miR-486	human, mouse	(19) (20)

References

1. Cao P, Feng Y, Deng M, Li J, Cai H, Meng Q, et al. MiR-15b is a key regulator of proliferation and apoptosis of chondrocytes from patients with condylar hyperplasia by targeting IGF1, IGF1R and BCL2. Osteoarthritis and cartilage. 2019;27(2):336-46. Epub 2018/12/07. doi: 10.1016/j.joca.2018.09.010. PubMed PMID: 30521861.
2. Wang J, Liu H, Tian L, Wang F, Han L, Zhang W, et al. miR-15b Inhibits the Progression of Glioblastoma Cells Through Targeting Insulin-like Growth Factor Receptor 1. Hormones & cancer. 2017;8(1):49-57. Epub 2016/11/30. doi: 10.1007/s12672-016-0276-z. PubMed PMID: 27896672.
3. Zhong Z, Li F, Li Y, Qin S, Wen C, Fu Y, et al. Inhibition of microRNA-19b promotes ovarian granulosa cell proliferation by targeting IGF-1 in polycystic ovary syndrome. Molecular medicine reports. 2018;17(4):4889-98. Epub 2018/01/25. doi: 10.3892/mmr.2018.8463. PubMed PMID: 29363717; PubMed Central PMCID: PMCPMC5865948.
4. Go H, Jang J-Y, Kim P-J, Kim Y-G, Nam SJ, Paik JH, et al. MicroRNA-21 plays an oncogenic role by targeting FOXO1 and activating the PI3K/AKT pathway in diffuse large B-cell lymphoma. Oncotarget. 2015;6(17).
5. Bai L, Liang R, Yang Y, Hou X, Wang Z, Zhu S, et al. MicroRNA-21 Regulates PI3K/Akt/mTOR Signaling by Targeting TGFβI during Skeletal Muscle Development in Pigs. PloS one. 2015;10(5):e0119396. doi: 10.1371/journal.pone.0119396.
6. Sahay S, Tiwari P, Pandey M, Gupta KP. PI3K/Akt Pathway and miR-21 are Involved in N-Ethyl-N-Nitrosourea-Induced F1 Mouse Lung Tumorigenesis: Effect of Inositol Hexaphosphate. Journal of environmental pathology, toxicology and oncology : official organ of the International Society for Environmental Toxicology and Cancer. 2019;38(1):69-81. Epub 2019/02/27. doi: 10.1615/JEnvironPatholToxicolOncol.2018026684. PubMed PMID: 30806292.
7. Lerman G, Avivi C, Mardoukh C, Barzilai A, Tessone A, Gradus B, et al. MiRNA Expression in Psoriatic Skin: Reciprocal Regulation of hsa-miR-99a and IGF-1R. PloS one. 2011;6(6):e20916. doi: 10.1371/journal.pone.0020916.
8. Geng Y, Sui C, Xun Y, Lai Q, Jin L. MiRNA-99a can regulate proliferation and apoptosis of human granulosa cells via targeting IGF-1R in polycystic ovary syndrome. Journal of assisted reproduction and genetics. 2019;36(2):211-21. Epub 2018/10/31. doi: 10.1007/s10815-018-1335-x. PubMed PMID: 30374732; PubMed Central PMCID: PMCPMC6420594.
9. Cheng H, Xue J, Yang S, Chen Y, Wang Y, Zhu Y, et al. Co-targeting of IGF1R/mTOR pathway by miR-497 and miR-99a impairs hepatocellular carcinoma development. Oncotarget. 2017;8(29):47984-97. Epub 2017/06/19. doi: 10.18632/oncotarget.18207. PubMed PMID: 28624790; PubMed Central PMCID: PMCPMC5564620.
10. Xie T, Zhang J, Kang Z, Liu F, Lin Z. miR-101 down-regulates mTOR expression and attenuates neuropathic pain in chronic constriction injury rat models. Neuroscience research. 2020;158:30-6. Epub 2019/09/19. doi: 10.1016/j.neures.2019.09.002. PubMed PMID: 31526851.

11. Zhang S, Wang M, Li Q, Zhu P. MiR-101 reduces cell proliferation and invasion and enhances apoptosis in endometrial cancer via regulating PI3K/Akt/mTOR. Cancer biomarkers : section A of Disease markers. 2017;21(1):179-86. Epub 2017/10/31. doi: 10.3233/cbm-170620. PubMed PMID: 29081412.
12. Garofalo M, Di Leva G, Romano G, Nuovo G, Suh S-S, Ngankeu A, et al. miR-221&222 Regulate TRAIL Resistance and Enhance Tumorigenicity through PTEN and TIMP3 Downregulation. Cancer Cell. 2009;16(6):498-509. doi: https://doi.org/10.1016/j.ccr.2009.10.014.
13. Hao J, Zhang C, Zhang A, Wang K, Jia Z, Wang G, et al. miR-221/222 is the regulator of Cx43 expression in human glioblastoma cells. Oncology reports. 2012;27(5):1504-10. Epub 2012/02/02. doi: 10.3892/or.2012.1652. PubMed PMID: 22294051.
14. Zhang Y, Sun Y, Peng R, Liu H, He W, Zhang L, et al. The Long Noncoding RNA 150Rik Promotes Mesangial Cell Proliferation via miR-451/IGF1R/p38 MAPK Signaling in Diabetic Nephropathy. Cellular Physiology and Biochemistry. 2018;51(3):1410-28. doi: 10.1159/000495590.
15. Fang X, Shen F, Lechauve C, Xu P, Zhao G, Itkow J, et al. miR-144/451 represses the LKB1/AMPK/mTOR pathway to promote red cell precursor survival during recovery from acute anemia. Haematologica. 2018;103(3):406-16. Epub 12/21. doi: 10.3324/haematol.2017.177394. PubMed PMID: 29269522.
16. Fornari F, Milazzo M, Chieco P, Negrini M, Calin GA, Grazi GL, et al. MiR-199a-3p regulates mTOR and c-Met to influence the doxorubicin sensitivity of human hepatocarcinoma cells. Cancer Res. 2010;70(12):5184-93. Epub 2010/05/27. doi: 10.1158/0008-5472.can-10-0145. PubMed PMID: 20501828.
17. Wang Y, Luo J, Wang X, Yang B, Cui L. MicroRNA-199a-5p Induced Autophagy and Inhibits the Pathogenesis of Ankylosing Spondylitis by Modulating the mTOR Signaling <i>via</i> Directly Targeting Ras Homolog Enriched in Brain (Rheb). Cellular Physiology and Biochemistry. 2017;42(6):2481-91. doi: 10.1159/000480211.
18. Liu Y, Lu C, Fan L, Wang J, Li T, Liu Z, et al. MiR-199a-5p Targets ZEB1 to Inhibit the Epithelial-Mesenchymal Transition of Ovarian Ectopic Endometrial Stromal Cells Via PI3K/Akt/mTOR Signal Pathway In Vitro and In Vivo. Reproductive sciences (Thousand Oaks, Calif). 2020;27(1):110-8. Epub 2020/02/13. doi: 10.1007/s43032-019-00016-5. PubMed PMID: 32046378.
19. Peng Y, Dai Y, Hitchcock C, Yang X, Kassis ES, Liu L, et al. Insulin growth factor signaling is regulated by microRNA-486, an underexpressed microRNA in lung cancer. Proceedings of the National Academy of Sciences of the United States of America. 2013;110(37):15043-8. Epub 2013/08/28. doi: 10.1073/pnas.1307107110. PubMed PMID: 23980150; PubMed Central PMCID: PMCPMC3773758.
20. Zhu M, Yi M, Kim CH, Deng C, Li Y, Medina D, et al. Integrated miRNA and mRNA expression profiling of mouse mammary tumor models identifies miRNA signatures associated with mammary tumor lineage. Genome Biol. 2011;12(8):R77. Epub 2011/08/19. doi: 10.1186/gb-2011-12-8-r77. PubMed PMID: 21846369; PubMed Central PMCID: PMCPMC3245617.
